# Evaluation of the Potential for Genomic Selection to Improve Spring Wheat Resistance to Fusarium Head Blight in the Pacific Northwest

**DOI:** 10.3389/fpls.2018.00911

**Published:** 2018-07-03

**Authors:** Haixiao Dong, Rui Wang, Yaping Yuan, James Anderson, Michael Pumphrey, Zhiwu Zhang, Jianli Chen

**Affiliations:** ^1^College of Plant Sciences, Jilin University, Changchun, China; ^2^Department of Crop and Soil Sciences, Washington State University, Pullman, WA, United States; ^3^Department of Plant Sciences, University of Idaho, Aberdeen, ID, United States; ^4^Department of Agronomy and Plant Genetics, University of Minnesota, St. Paul, MN, United States

**Keywords:** wheat, fusarium head blight, genomic selection, disease resistance, cross validation, molecular breeding, cultivars, Pacific Northwest

## Abstract

Fusarium Head Blight (FHB) has emerged in spring wheat production in Pacific Northwest during the last decade due to factors including climate changes, crop rotations, and tillage practices. A breeding population with 170 spring wheat lines was established and screened over a 2-year period in multiple locations for FHB incidence (INC), severity (SEV), and deposition of the mycotoxin, deoxynivalenol (DON). A genome-wide association study suggested that the detectable number of genetic loci and effects are limited for marker-assisted selection. In conjunction with the success of breeding on FHB resistance in other programs, genomic selection (GS) was suggested as a better option. To evaluate the prediction accuracy of GS in the current breeding population, we conducted a variety of validations by varying proportions of testing populations and cohorts based on both FHB resistance and market class, including soft white spring (SWS), hard white spring (HWS), and hard red spring (HRS). We found that INC had higher heritability, higher correlation across years and locations, and higher prediction accuracy than SEV and DON. Prediction accuracy varied among the scenarios that restricted the testing population to a certain cohort. For a small set of newly developed or introduced lines (<17), prediction accuracy will be about 60% if the lines have similar genetic relationships as those among the current 170-line training population. However, we expect a lower prediction accuracy if new lines are selected for a specific characteristic, such as FHB resistance or market class. With the exception of DON in the SWS lines, the current training population is capable of making reasonably accurate predictions for FHB-resistant lines in most of the major market classes. For SWS, adding more lines or further phenotyping is required to improve prediction accuracy. These results demonstrate the potential and challenges of GS, especially for developing FHB-resistant varieties in the SWS market class.

## Introduction

Fusarium head blight (FHB) is a devastating disease that affects corn and small grain crops such as wheat in humid conditions (Schroeder and Christensen, 1963; Wang et al., 1982; Snijders, 1990). FHB causes shriveled kernels, significant yield losses, and deposition of the mycotoxin, deoxynivalenol in the infected seeds, which renders the grain unsuitable for human consumption and animal feed (McMullen et al., 1997). Historically, during the last three decades, FHB has been an endemic disease in the north-central and eastern regions of the United States. However, in recent years, the disease has emerged in the Pacific Northwest (PNW), coinciding with increased corn production, reduced tillage farming practices, and changing climate in the region (Marshall et al., 2012). Due to higher temperatures and greater humidity during flowering time in the PNW, spring wheat is more susceptible to FHB than winter wheat. Most of the wheat cultivars currently grown in the PNW are susceptible to FHB and, when infected, often produce high levels of deoxynivalenol (Marshall, 2014). Therefore, development of FHB-resistant wheat cultivars for the region is critically needed to reduce the impending negative consequences, including reduced crop yield and quality and increased costs for fungicides.

The traditional genetic marker-based breeding method is called marker-assisted selection, which utilizes known, previously identified quantitative trait loci (QTL)-associated markers. In the last two decades, more than 200 FHB-resistant QTL have been identified across the entire wheat genome (Buerstmayr et al., 2009; Liu et al., 2009). However, none of these QTL confer promising resistance to FHB. For example, although one of the major QTL, *Fhb1* identified on chromosome 3BS (Anderson et al., 2001; Liu and Anderson, 2003) could reduce disease occurrence by 20–25% (Pumphrey et al., 2007), this QTL was originally identified from the Chinese line, Sumai 3, and the favorable allele has a very low frequency in North American germplasm (Sneller et al., 2010; Bernardo et al., 2012). Introducing this allele may not only take time, but also risk the diversity among North America germplasm. Additional QTL were also confirmed on chromosomes 2D (Shen et al., 2003a; Yang et al., 2005; Cuthbert et al., 2007), 3A (Shen et al., 2003a; Steiner et al., 2004; Yang et al., 2005), 5AS (Buerstmayr et al., 2003; McKendry et al., 2004; Chen et al., 2006), and 6B (Shen et al., 2003b; Yang et al., 2005; Cuthbert et al., 2007). Similar to *Fhb1*, most of these QTL were originally derived from Chinese wheat germplasm.

Efforts to identify FHB resistance in native North American winter wheat germplasm have been undertaken (Sneller et al., 2010; Arruda et al., 2016). Both Sneller et al. (2010) and Arruda et al. (2016) showed that FHB resistance is conferred by many small effect QTL in soft red winter wheat lines grown in the eastern United States. A research has been conducted on other native spring wheat germplasm without Sumai 3 backgrounds, which included 170 lines developed by wheat breeders at PNW and the International Maize and Wheat Improvement Center (CIMMYT) (Wang et al., 2017). This study also suggested that FHB resistance in spring wheat is controlled by a relatively large number of QTL with small effects. Thus, there is a critical need to assemble the total genetic effects of individual lines—accounting not only for QTL with large or moderate effects, but also QTL with small effects, which collectively control a large proportion of the total genetic variance. This method is commonly known as genomic selection (GS).

GS was introduced to plant breeding in 1994 to evaluate yield potential in maize inbred lines as the Best Linear Unbiased Prediction (BLUP), using restriction fragment length polymorphism (RFLP) markers covering the maize genome (Bernardo, 1994). The method is now known as genomic BLUP, or gBLUP. GS has been used to predict genetic merit in animals (Hayes et al., 2009; Guo et al., 2011) and plants (Heffner et al., 2009; Jannink et al., 2010). GS has also been applied in winter wheat programs for FHB resistance in the mid-western and eastern United States and Canada. Rutkoski et al. (2012) compared the accuracy of different GS models for FHB-related traits using 170 winter wheat lines from 18 different breeding programs and more than 2,000 diversity array technology markers and single-sequence repeat markers. Arruda et al. (2015) evaluated different factors affecting the accuracy of genomic prediction using 273 winter wheat breeding lines and 5,054 genotyping-by-sequencing markers. Both studies demonstrated that GS is a very promising breeding strategy for FHB resistance in winter wheat.

Our study aimed to fill the knowledge gaps on FHB in spring wheat by evaluating prediction accuracy of using the existing 170 lines under three conditions: (1) no restriction on testing cohort of market class and segmentation of FHB resistance; (2) restriction of testing cohort to market classes; and (3) restriction of testing cohort to segmentation of FHB resistance. This knowledge was intended to provide guidance for developing a GS pipeline for breeding FHB-resistant wheat in the PNW. Additionally, we expect the new knowledge and the approach used to benefit similar breeding programs in other traits, geographic areas, and breeding programs for other crops.

## Materials and Methods

### Plant Materials and Disease Evaluation

The spring wheat panel used in this study and the FHB disease evaluation were described in Wang et al. (2017). In short, a total of 170 spring wheat cultivars and elite lines developed from the breeding programs in the PNW and CIMMYT were used in this study. The 170 lines (PNW panel, hereafter) include 26 lines from Washington State University (WSU), 33 from University of Idaho (UI), 34 from University of California, Davis (UCD), 25 from Montana State University (MSU), 49 from CIMMYT, two from Limagrain Cereal Seeds (LCS), and one from SSK (Agriculture and Agri-Food Canada, Saskatchewan). Over 50% of lines have been used as parental lines in one or more breeding programs. Therefore, this PNW panel is representative of the genetic diversity in currently used germplasm. Based on the end-use products, the panel contains three main market classes of spring wheat: soft white spring (SWS), hard white spring (HWS), and hard red spring (HRS) wheat.

The phenotypes of the 170 lines were collected from three field nurseries [Saint Paul, MN (StP), Crookston, MN (CrK), and Aberdeen, ID (AB)] and one greenhouse (GH) over 2 years (2015 and 2016). Fields were divided into zones according to field orientation. Checks were placed in each zone for validation. The checks included the FHB resistant cultivars Alsen, BacUp, W14, and AC Barrie (Chen et al., 2006; Zhang et al., 2016) and the FHB susceptible cultivars Roblin, Wheaton, and UI Platinum (Chen and Marshall, 2012; Zhang et al., 2016). In MN fields, 190 lines, including the 170 lines that were genotyped and used in this study, were randomly assigned to two zones by using an un-replicated augmented complete block design. In each zone, 95 lines and five checks (Alsen, BacUp, W14, Roblin and Wheaton) were randomly assigned. In Aberdeen fields, the 190 lines were randomly assigned to five zones. In each zone, 38 lines and two checks (AC Barrie and UI Platinum) were randomly assigned with two replications. In GH, two checks (AC Barrie and UI Platinum) were used, and each line had four replications. The inoculum for StP and CrK nurseries was prepared by balanced mixing 30–39 *F. graminearum* isolates, as used in Zhang et al. (2016).

The inoculum used in AB nursery and GH experiment was isolated as single spore isolate from natural infected seeds in Idaho. This isolate is virulent to most wheat cultivars in Idaho. The plots in the StP and AB nurseries were individually sprayed two to three times with the prepared inoculum using a CO_2_-pressure backpack sprayer. The plants in the GH experiment were point inoculated with approximately 5 μl of macroconidia suspension at the concentration of 8–10 × 10^4^ ml^-1^ using a pipette dropper. For both methods (spray and point inoculation), to facilitate even distribution and adherence of fungal spores on the plant, a surfactant (Tween 20: polyoxyethylene-20-sorbitan monolaurate) was added (100 μl per liter) to the inoculum suspension before use. Corn spawn method was applied to the CrK nursery. The colonized corn grains were spread throughout the nursery twice at the wheat jointing stage and at 1 week later. For all nurseries and GH experiments, the plants were misted immediately after inoculation to promote infection.

Due to variable disease pressure, disease assessment was performed at 21 days after inoculation in the StP and CrK experiment locations, and at 28 days after inoculation in the AB and GH experiment locations. Incidence (INC), severity (SEV), and deoxynivalenol concentration (DON) were assessed as FHB disease reactions. INC was recorded as the percentage of infected spikes in a headrow plot. SEV was visually diagnosed as the percentage of infected spikelets in each spike. DON (ppm) was measured using grain samples from 30 randomly selected heads for each line in the StP and CrK field nurseries and from the composite of all plants in the AB nursery (Supplementary Table S1). The procedure to quantify DON is described in Fuentes et al. (2005) and Mirocha et al. (1998) and briefly summarized in Wang et al. (2017). In short, the sample was prepared and passed through a column and then the dried filtrate was derivatized for gas chromatography-mass spectrometry (GC/MS) analysis. The characteristic ions of DON with fragment ion (m/z value) of 235.10 was detected as target while the fragment ions of 259.10 and 422.10 as reference ions.

### Phenotypic Data Analysis

In this study, we treated each year-location combination as an environment. We evaluated FHB SEV in five environments and assessed FHB INC and DON in four environments. In each environment, the raw phenotypic values within replicates were adjusted by using a spatial analysis. The analyses were performed by using R package “lme4” (Bates et al., 2015) with zone fitted as random effect. For the phenotypic data collected from GH, no spatial adjustment was applied. The arithmetic mean of all replications in each trial was calculated and used as the single environment phenotypic data value. We applied R package “lme4” to estimate the BLUP values for the three phenotypes (INC, SEV, and DON) across different environments (Supplementary Table S1). The lines and environments were considered random effects in the mixed model. Raw phenotypes, means and BLUPs were displayed by the pairs.panel function in R package “psych” (Revelle, 2017) to illustrate the distributions with trends indicated by the correlations ellipse.

### Genotypic Markers

Of the total 170 lines in the PNW panel, 143 belong to the Spring Wheat Association Mapping (SWAM) panel developed by Triticeae Coordinated Agricultural Project (T-CAP). The Illumina 90K genotypic markers for these 143 lines were downloaded from the Triticeae Toolbox (T3) website^1^ operated by T-CAP. The remaining 27 lines were also genotyped with the Illumina 90K SNPs assay at the USDA-ARS Cereal Crops Research Unit, Fargo, ND and the allele calls were performed with GenomeStudio v2011.1 (Illumina Inc., Hayward, CA, United States). Finally, a total of 11,523 common SNP markers were selected by combining the two sets of genotyping data. Genetic positions for the selected SNP markers were retrieved from the consensus map for 90K SNP markers developed by Wang et al. (2014). After filtering out SNP markers with a missing rate of more than 10% or with a minor allele frequency (MAF) less than 0.05, 10,101 SNPs were retained for further analysis. The missing genotypes were imputed using the java package “LinkImpute” (Money et al., 2015), based on a *k*-nearest neighbor genotype imputation method, LD-kNNi (Troyanskaya et al., 2001), which is designed for unordered markers.

### Principal Component (PC) Analysis

Principal Component analysis was conducted with all genetic markers by using the prcomp function in R (R Core Team, 2017). We used the default (correlation) option to derive eigen values and eigen vectors, which implied that all genetic markers were weighted the same. The markers with large MAFs contributed the same as markers with small MAFs. The first three PCs were used to present the population structure categorized by market classes and origins. The first three PCs were also used as the fixed effects for estimation of heritability and genomic prediction.

### Estimation of Heritability

The observations in each environment, means and BLUPs across environments, were the response variables in a fixed and random effects mixed linear model. The fixed effects were the first three PCs. The random effects were the total additive genetic effects of individuals in addition to the residuals. The variance and covariance matrix of the total additive genetic effects was defined as the product of the additive relationship matrix derived from markers (Zhang et al., 2007) and the genetic variance. The statistical model can be written as follows:

y=μ+Xβ+Zu+ϵ

where *y* is a vector (*n* × 1) of observations with n as number of lines; μ is the overall mean; β is a vector (*p* × 1) of fixed effects with *p* as the number of fixed effects, specifically, the first three PCs derived from all markers (*p* = 3); *X* is a design matrix (*n* × *p*) for fixed effects; u is a vector (*n* × 1) of random effects of the total additive genetic effects of individuals; *Z* is a design matrix (*n* × *n*) for random effects *u*; and ε is the residuals. The random effects follow normal distributions: *u*∼*N*(0, *K*σ_u_^2^), ε∼*N*(0, *I*σ_ε_^2^), where *I* is the identity matrix and *K* is the additive relationship matrix calculated from all genetic markers by using VanRaden algorithm (VanRaden, 2008). The matrix was rescaled to pedigree-like relationship matrix, implemented in GAPIT (Lipka et al., 2012; Tang et al., 2016). The rescaled relationship matrix had the maximum elements of 2 on the diagonals and the minimum element of 0 off diagonal for the two individuals that were the least related; σ_u_^2^ and σ_ε_^2^, are the variance of individual additive genetic effects and variance of residuals, respectively. The estimations of the genetic and residual variances were conducted by using the EMMA algorithm (Kang et al., 2008).

### Genomic Prediction

Ridge regression was employed to perform genomic prediction by using mixed.solve function implemented in R package “rrBLUP v4.5” (Endelman, 2011). This statistical model has the same format as the model to estimate heritability (1). However, matrix *Z* and vector *u* are defined differently. *Z* is a design matrix (*n* × *m*) for random effects, specifically, the matrix for genotypes; *u* is a vector (*m* × 1) of random effects of markers with m as number of markers. The random effects of markers follow normal distributions: *u*∼*N*(0, *I*σ_u_^2^), where *I* is the identity matrix and σ_u_^2^ is the variance of individual marker genetic effects.

### Evaluation of Prediction Accuracy

Monte Carlo cross-validations (Xu and Liang, 2001) were employed to evaluate accuracy of prediction by using rrBLUP. A proportion, varying from 10 to 90%, of lines were randomly selected as the testing population and the remaining lines as the training population. Both the genotypes and the observed phenotypes in the training population were used to estimate the effects of genetic markers. The estimated marker effects and the genotypes of the testing population were used to calculate the predicted phenotypes, which also included the estimated fixed effects of the first three PCs. The observed phenotypes of the testing population were only used to calculate the Pearson correlation coefficient between the observed and the predicted phenotypes. The stochastic process was replicated 1,000 times and the prediction accuracy distributions, means, and standard errors were reported.

Selection of testing populations and training populations was also restricted to specific cohorts of the spring wheat lines. The first scenario had no restriction; all 170 lines were treated homogeneously. The second scenario restricted the testing population to either FHB-resistant lines or FHB-susceptible lines. The third scenario restricted the testing population to the lines from a specific market class. We also created some scenarios that restricted both the training and testing populations. For example, when we restricted the testing population to SWS, we also restricted the training population to SWS.

### Data Availability

Genotypes, phenotypes and the classification information (market classes, origins, and FHB susceptibility) of 170 wheat lines are available in Supplementary Table S1.

## Results

**FIGURE 1:**
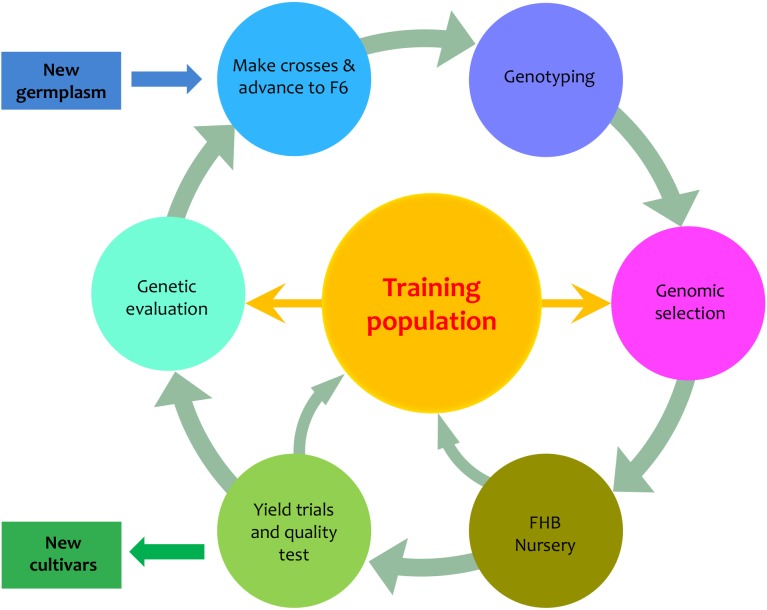
Proposed PNW FHB genomic breeding pipeline. The pipeline is centralized by the training population which currently contains 170 lines from Pacific Northwest (PNW) and the International Maize and Wheat Improvement Center (CIMMYT). The 170 lines have been assessed for FHB resistance in two different states (Idaho and Minnesota) over 2 years (2015 and 2016). The training population will be expanded from both internal and external germplasm. The external germplasm will include the lines with genotypes and phenotypes on FHB resistance. The internal germplasm includes the newly developed F6, F7, and F8 lines that will be genotyped and phenotyped for FHB resistance, grain yield, and end-use quality. The outputs of the pipeline are lines with FHB resistance and good agronomic performance.

We designed a PNW FHB genomic breeding pipeline that includes six elements in addition to input and output (**Figure 1**). Central to the pipeline are the 170 PNW and CIMMYT spring wheat lines, which are used as the primary training population to develop new varieties with FHB resistance. The input is the newly introduced germplasm. The output is the newly developed varieties. The six surrounding elements of the pipeline are advanced breeding lines (F6), genotyping, GS, FHB nursery, field trials, and genetic evaluation. GS is used to assess new genotypes and genetic evaluation is used to assess new phenotypes. Both the FHB nursery and field trials focus on phenotyping, but the FHB nursery evaluates FHB resistance and the field trials evaluate agronomic traits such as grain yield and quality. Our primary interest was to determine how accurately we could predict the newly developed or the newly introduced varieties to our existing training population. We investigated the phenotypic correlation across environments, trait heritability under each environment and heritability of mean across environments, and prediction accuracy through validation.

### Phenotypic Correlation Across Locations and Years

**FIGURE 2:**
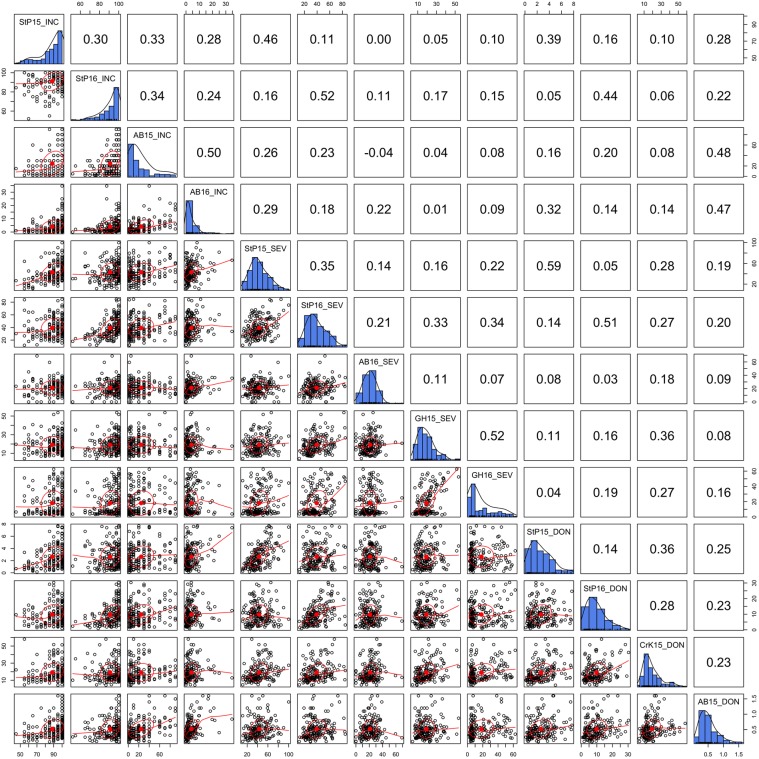
Correlation and distribution of FHB phenotypes at different environments. The three FHB traits studied were incidence (INC), severity (SEV), and deoxynivalenol concentration (DON). The environments were defined as combinations of year and location. The study locations were StP (Saint Paul, MN, United States), CrK (Crookston, MN, United States), AB (Aberdeen, ID, United States), and greenhouse (GH). Years included 2015 (15) and 2016 (16). Environments are labeled on the diagonals. The diagonals also illustrate the distribution of the means across replicates within each environment. The dots on the scatterplots off the diagonals represent the mean across replicates. The red line is the robust fitting using lowess regression, the red dot and the circle represent the correlation ellipse.

Three FHB-related traits, INC, SEV, and DON, were measured in two to four locations during a 2-year period. The distributions of the three traits under different environments and their BLUPs were demonstrated in our previous study (Wang et al., 2017). In this study, we detailed the phenotypic correlation across environments with scatter plots and Pearson correlations (**Figure 2**). As shown by the distributions on the diagonals, DON and SEV exhibit close to a normal distribution. However, the INC distribution is close to the Poisson distribution. The side of tails varied across environments for the Poisson distribution. Two of them were on the left and one on the right. Nevertheless, INC had the highest correlation, ranging from 0.24 to 0.5 with mean of 0.33. SEV had the lowest correlations across environments, ranging from 0.07 to 0.52 with mean of 0.25. In general, the correlations were low for all three traits. This finding suggests that FHB must be evaluated with multiple locations and multiple years to achieve reliable mean values.

### Similarity Between Mean and BLUP

To examine prediction accuracy through validation, the training population and testing population should be completely separate so that the phenotypes of the testing population are not used as the training data. Means across the environments straightly satisfies this requirement. Although BLUP is derived in a mixed linear model with individual lines treated as uncorrelated, BLUPs are potentially dependent among lines due to adjustments for the effects of location, year, and replicate. In our previous gene mapping study (Wang et al., 2017), BLUPs were used as the response variable, instead of means. In this study, we wanted to know how similar the mean is to BLUP.

**FIGURE 3:**
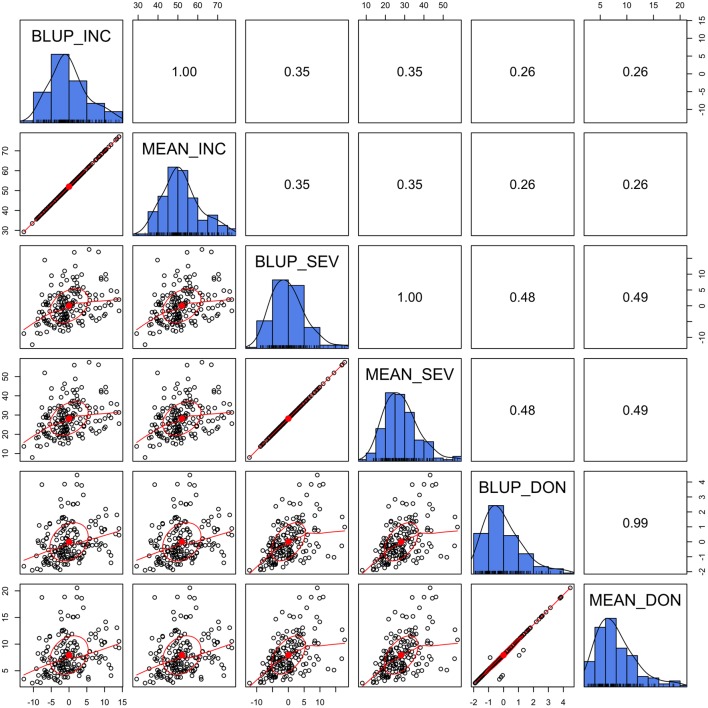
Alignment between mean and Best Linear Unbiased Prediction (BLUP). The three FHB traits studied were INC, SEV, and DON. The mean was calculated as the mean across replicates, locations, and years. BLUP was calculated in our previous study (Wang et al., 2017) by using mixed linear model with lines treated as unrelated. The diagonally oriented bar graphs illustrate the distribution of the mean and BLUP values for each phenotype. Displayed below the diagonal, are the scatter plots for mean and BLUP values; displayed above the diagonal are their Pearson correlation values. The red line is the lowess regression fitting curve; the red dot and circle construct the correlation ellipse.

We demonstrated that the BLUP was almost identical to the mean, especially for INC and SEV, where the data were nearly balanced and missing data were minimal (**Figure 3**). In the case of lines treated as unrelated, the BLUP is identical to the mean. Even for DON, BLUPs were almost identical to the means except for a few lines with missing values. The BLUPs of these lines equal their means adjusted by the means of the other lines. Using the BLUP of the training population involves the phenotypes of the testing population. Therefore, we chose to use means to evaluate prediction accuracy through validation.

### Market Classes and Origin of Lines in Relation to PC Analysis

Market class is a major characteristic in wheat breeding. Three major classes of spring wheat are grown in the PNW. HRS and HWS wheat are used for different end-use products compared to SWS wheat. Therefore, HRS and HWS differ from SWS in important requirements such as protein content, flour hardness, and other quality traits. Among the 170 lines we studied, 79 were HWS lines, 65 were HRS, and 24 were SWS. The remaining two lines were soft red spring (SRS) wheat. The 170 lines were from seven origins. The top five major origins (UCD, UI, WSU, MSU, and CIMMYT) contributed 26–49 lines each and 167 in total. The remaining lines consisted of two from LCS and one from SSK (Supplementary Table S1).

**FIGURE 4:**
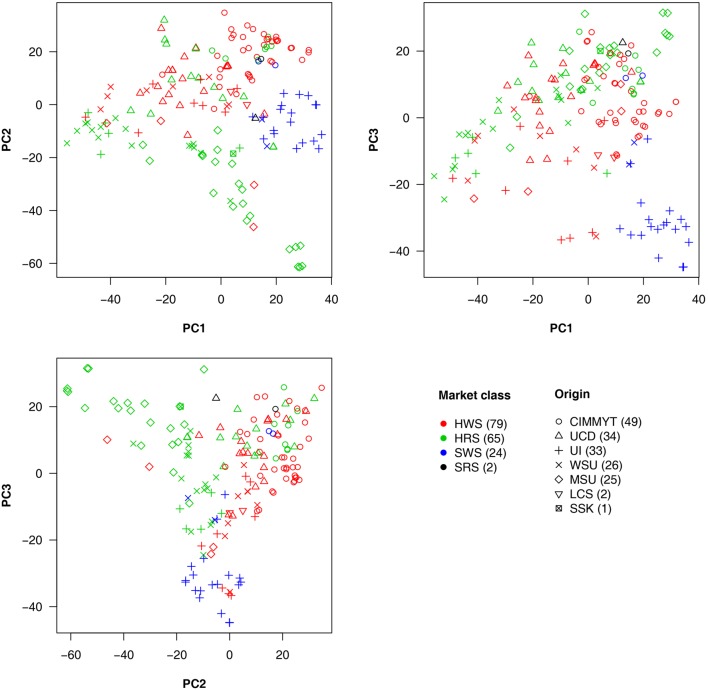
Wheat line origins and market classes revealed by principal component analysis. The principal components (PCs) were calculated by using all genetic markers. The first three PCs explained 7.4, 6.2, and 4.9% of the total variation, respectively. The wheat market classes studied were Hard Red Spring (HRS), Hard White Spring (HWS), Soft White Spring (SWS), and Soft Red Spring (SRS). The top five major origins for most of the lines are UCD (University of California, Davis); UI (University of Idaho); WSU (Washington State University); MSU (Montana State University); and CIMMYT (the International Maize and Wheat Improvement Center) plus two lines from LCS (Limagrain Cereal Seeds) and one line from SSK (Agriculture and Agri-Food Canada, Saskatchewan). Numbers in the brackets in legend represent the amount of lines in each category.

To explore the relationship among lines, origins, and market classes, PCs were derived from all available SNPs. The first three PCs explained around 20% of the total variance. Pairwise relationships for the 170 lines are illustrated in **Figure 4** with market classes and origins indicated by colors and shapes, respectively. The SWS market class stands out from the other two classes on all three plots. Substantial overlap occurs between HRS and HWS. Most of the lines with the same origin were clustered. For example, the lines from CIMMYT (circles) are centered in the upper half of the PC1 vs. PC2 plot. The lines from MSU (diamonds) are concentrated in the top left of the PC2 vs. PC3 plot. We found a strong association between origins and market classes. Almost all the individuals from UI are SWS, those from MSU are mostly HRS, and those from CIMMYT are mostly HWS.

### Heritability Estimation

**FIGURE 5:**
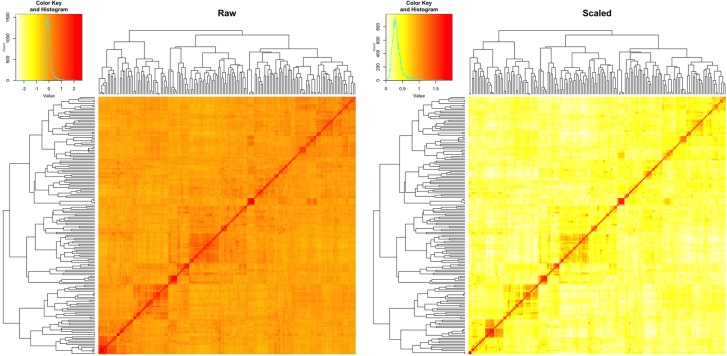
Rescale of the relationship matrix into the kinship-like matrix. The raw matrix (left) was obtained from 10,101 SNPs for 170 spring wheat lines by using VanRaden algorithm, implemented in GAPIT. After rescale by using the transformation in GAPIT, the range of elements in the matrix fell into the same range as the kinship matrix (right), from 0 to 2.

We estimated the narrow sense heritability by using a fixed effect and random effect mixed linear model. Besides the residual effects, the random effects are the individual total additive genetic effects with variance structure defined by an additive relationship matrix. The raw additive relationship matrix was derived by using the VanRaden algorithm implemented in GAPIT. Most of the elements in the matrix were close to zero, ranging from -3 to 3 (**Figure 5**). The estimation of heritability requires the minimum number of elements to be zero for uncorrelated individuals and the maximum number of elements to be two on the diagonals for inbred individuals. Thus, we conducted the pedigree kinship-like transformation, implemented in GAPIT. The scaled relationship matrix had desirable features to estimate heritability (**Figure 5**).

**FIGURE 6:**
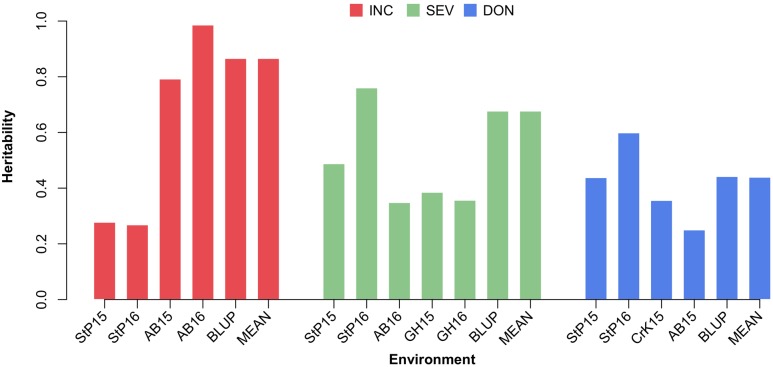
Estimate of heritability of three FHB traits. The three FHB traits studied were INC, SEV, and DON. Heritability was estimated for these traits under each environment, and using the mean and BLUP across the environments. Environment was defined as the combination between location and year. The four locations were Saint Paul, MN (StP), Crookston, MN (CrK), Aberdeen, ID (AB), and one GH, studied over 2 years (2015 and 2016).

The variances of individuals’ total additive genetic effects and residual effects were estimated by using the EMMA algorithm. The total phenotypic variance was defined as the sum of the additive genetic variance and the residual variance. The proportion of the additive genetic variance over the total variance was defined as the narrow sense heritability. The heritability estimates for phenotypes measured under different environments, the means across environments, and BLUP are illustrated in Supplementary Table S2, Supplementary Figure S1, and **Figure 6**. Overall, the estimated heritabilities of INC were much higher than SEV and DON. This finding agrees with the finding that the phenotypes of INC were more correlated among environments.

### Validation With a Homogenous Cohort

**FIGURE 7:**
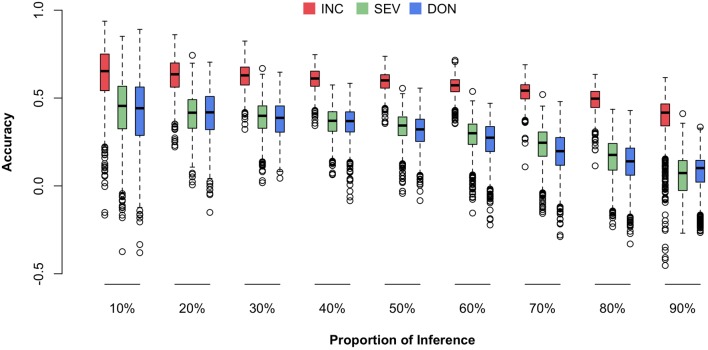
Prediction accuracies for three FHB traits under different fold of cross-validation. The three traits studied were INC, SEV, and DON. The cross-validation was performed by randomly selecting 10–90% of the lines from the total 170 lines and using that proportion as the testing population. The remaining lines were used as the training population. The prediction accuracy was calculated as the Pearson correlation between the observed and predicted phenotypes.

We were particularly interested in prediction accuracy when crosses among our current 170-line training population were advanced to higher generations (e.g., F6–F8) or when similar new external lines were introduced. To fill this knowledge gap, we randomly masked the phenotypes (i.e., means) of a certain proportion of the 170 lines and treated the masked lines as the future testing population (inference). The remaining lines were treated as the training population (reference). The genotypes of individuals in both the training and testing populations were used to calculate the Genomic Estimated Breeding Values (GEBVs) for all individuals, including those in the training and testing populations. However, only the phenotypes of individuals in the training population were used to estimate the effects of all markers by using the rrBLUP package in R. The prediction accuracy was calculated as the Pearson correlation between the predicted and the observed phenotypes. The predicted phenotypes were the sum of GEBV and the fixed effects, the first principal components. The randomization was replicated 1,000 times and the distributions, means, and standard deviations of the prediction accuracies were reported (**Figure 7** and Supplementary Table S3).

Consistent to the higher correlation across environments and higher heritability for INC compared to the other traits, prediction accuracy was also higher for INC. Prediction accuracy also depended on training population size. The scenario with 90% of 170 lines as the testing population had the lowest prediction accuracy. The scenario with 10% of the lines remaining as the testing population had the highest prediction accuracy. In practice, this latter scenario is the most useful because it uses almost all of the currently available resources if the number of introduced new lines is less than 17 and they have similar relationships with the 170 lines. In this case, the prediction accuracy is about 0.65 for INC and 0.45 for both SEV and DON.

### Validation With a Resistant Cohort

**FIGURE 8:**
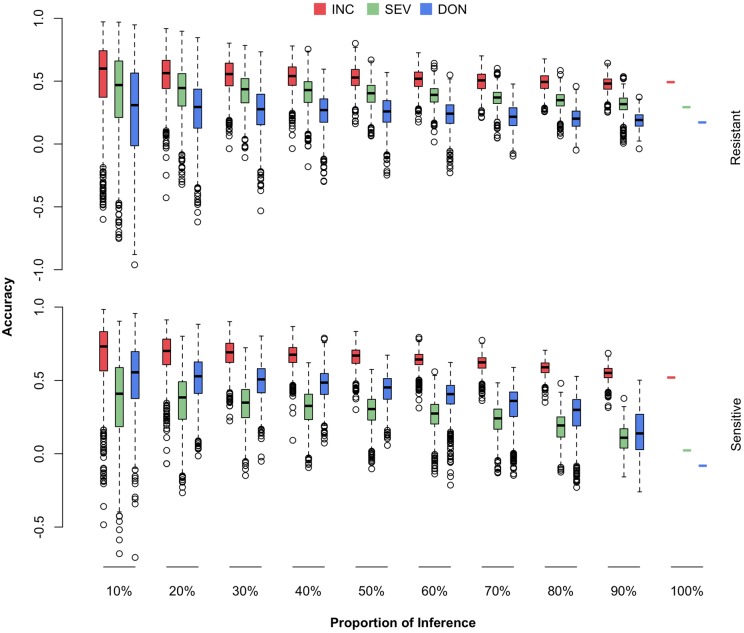
Prediction accuracies of FHB traits when different proportions of FHB-resistant or FHB-susceptible lines were used as the testing population. The 170 wheat lines were classified into resistant (69) and susceptible (101) subpopulations. Both subpopulations were randomly sampled and used as the testing population with proportions ranging from 10 to 100%. The three traits studied were INC, SEV, and DON. The prediction accuracy was calculated as the Pearson correlation between the observed and the predicted phenotypes.

The newly developed varieties, or the new lines introduced to the current breeding population, are most likely to be resistant to FHB. The most critical question is how well a certain amount of such lines can be predicted. Among the 170 lines in the current breeding population, 69 were identified by the breeders (Dr. Jianli Chen and Dr. Michael Pumphrey) as resistant lines and the rest as susceptible lines (Supplementary Table S1 and Supplementary Figure S2). For each subpopulation, we randomly masked a certain proportion of the lines (10–100%) as the testing population. The remaining lines, including all the lines from the opposite subpopulation, were treated as the training population. The randomization was also replicated 1,000 times. The distributions, means, and standard deviations of the prediction accuracies were reported (**Figure 8** and Supplementary Table S4).

We observed that prediction accuracies for the resistant lines were less sensitive to the proportion of resistant lines removed from the training population, compared with prediction accuracies for the susceptible lines as the proportion of susceptible lines were removed. This finding suggests that the current FHB-susceptible lines are valuable for predicting the resistant lines. Especially for the two low heritability traits, SEV and DON, the prediction accuracies for susceptible lines dropped almost to zero when the training population contained only the resistant lines. In contrast, the prediction accuracies for the resistant lines remained at 50, 35, and 20% for INC, SEV, and DON, respectively, even when the training population contained susceptible lines only.

We also observed that prediction accuracy was much lower for the scenario that restricted the testing population to resistant lines than the scenario with a homogeneous cohort. For example, a homogeneous cohort with 90% of lines as the training population resulted in a prediction accuracy of 0.63 for INC. In contrast, a resistant cohort that retained all susceptible lines and 90% of resistant lines as the training population, resulted in a prediction accuracy of 0.53 for INC on resistant lines. This finding suggests that to prevent the overestimate of prediction accuracy as future testing population contains resistant lines only, not a mixture of both resistant and susceptible lines, breeding plans should be based on the scenarios that validate a resistant cohort, not scenarios with a homogeneous cohort.

### Validation With a Market Class Cohort

**FIGURE 9:**
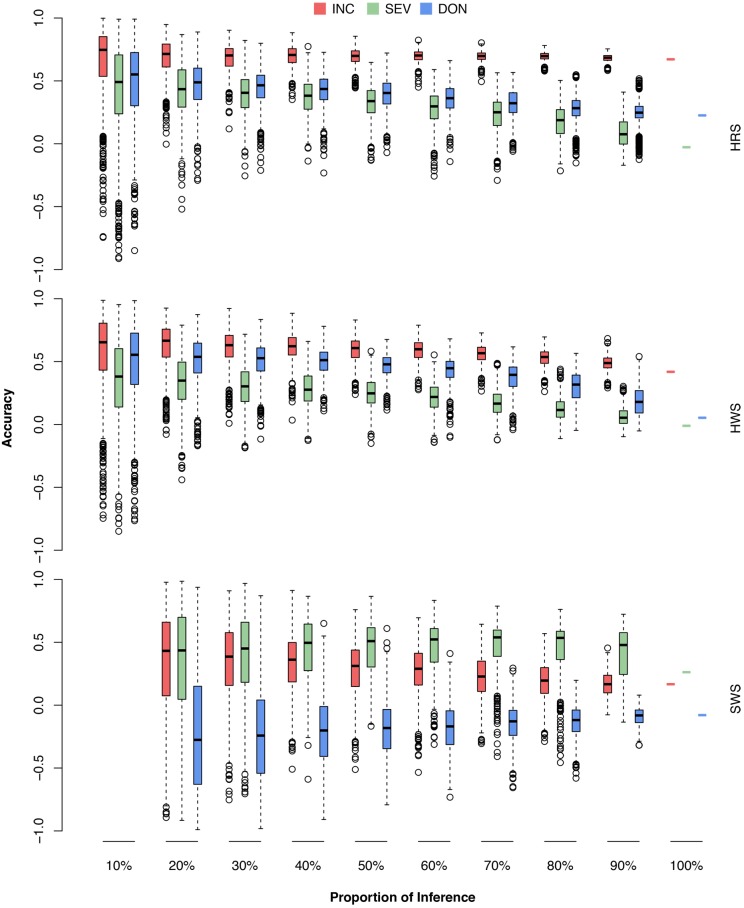
Prediction accuracies of FHB traits when different proportions of wheat market class lines were used as the testing population. The wheat market classes studied were HRS, HWS, and SWS. For each market class, 10–100% were randomly removed as the testing population; the rest of the lines, including lines from the other market classes, were used as the training population. The three FHB traits studied were INC, SEV, and DON. The prediction accuracy was calculated as the Pearson correlation between the observed and the predicted phenotypes. The combination of 10% and SWS was not available because, in this scenario, only two lines were in the testing population. The Pearson correlation would be either –1 or 1.

Our wheat breeding program involves three major market classes (HRS, HWS, and SWS) for different end-use products (Supplementary Table S1). The knowledge gap is how the prediction accuracy relates to the amount of new lines from a particular market class. To fill this gap, we randomly masked a certain proportion (10–100%) of lines from a particular market class into the testing population and used the remaining lines, mainly from other market classes, as the training population. The randomization was also replicated 1,000 times. The distributions, means, and standard deviations of the prediction accuracies were reported (**Figure 9** and Supplementary Table S5).

As demonstrated by the population structure from the PC plots (**Figure 4**), HRS and HWS are more connected to each other than to SWS. Consequently, the HRS lines can be predicted well from the HWS lines and vice versa. In contrast, the SWS lines cannot be predicted from the HRS or HWS lines; for example, the accuracies for DON were near or below zero. We narrowed down the problem by conducting the validation for each market class separately (Supplementary Figure S3 and Supplementary Table S6). In this scenario, all the lines in the training population were from the same market class as the lines in testing population. We observed a similar trend as the scenarios with training populations that contained lines from other market classes. The number of lines in SWS was almost one third of other two classes and almost all of them have the same origin. These are two possible reasons caused the low prediction in SWS.

## Discussion

In this study, various types of analyses and validations were performed to evaluate the potential of genomic prediction to improve resistance to FHB for spring wheat in the PNW. The procedures we used included determination of response variables, estimation of heritability, and validation of prediction accuracy under different scenarios. Although some of the procedures are specific to our own breeding program, other wheat breeding programs and even other crop breeding programs can also benefit from our methods and findings.

### Mean vs. BLUP

The validation of GS requires that the phenotypes of the testing population be used only for comparison of the prediction accuracy. In this study, we chose to use the mean for two reasons. First, we wanted to satisfy the requirement of validation that prevents the use of phenotypes in the testing population to predict themselves. Second, the mean and BLUP were highly identical in this study. The correlation between mean and BLUP was 100% for INC and SEV and 99% for DON. Thus, the results inferred from mean are robust enough to presume similar results in future GS based on BLUP.

### Marker-Based Kinship and Heritability Estimation

Heritability is defined as the proportion of genetic variance over the total variance, which is the sum of the genetic variance and the residual variance. Genetic variance and residual variance are usually estimated in a mixed linear model with genetic effect and residual effect as the random effects. The variance structure of the genetic effect is the relationship matrix derived from either pedigree or genetic markers. The matrix is twice of co-ancestry, which is the probability of identical by descent. Consequently, the minimum element is zero indicating no relationship. The maximum element is two for identical twins or an inbred with itself. The genetic variance estimated with such a relationship matrix is adjusted to the base genetic variance among founder who are completely uncorrelated. When the matrix is derived from pedigree, it satisfies all the requirements.

Many algorithms can be used to calculate the relationship matrix among individuals based on genetic markers, including Loiselle (Loiselle et al., 1995) and VanRaden. Most of these relationships do not have the properties corresponding to pedigree-based kinship. Consequently, the estimated genetic variances do not correspond to the genetic variance among individuals of the base population. The proportion to the total variance is artificially defined based on the selection of algorithms.

The 170 lines used in this study were inbred wheat. The diagonals of the relationship matrix should be two. If we assume the farthest two inbred lines are uncorrelated, their corresponding element should be the minimum, zero. With these assumptions, we applied a transformation, implemented in GAPIT, for the raw relationship matrix calculated by the VanRaden algorithm. After the transformation, the maximum element was 2 and the minimum element was 0. The maximum element was more likely to be correct than the minimum element. Therefore, interpretation of estimated heritability should be inferred with caution, even the pedigree-like relationship matrix used in this study.

### Breeding for FHB Resistance for SWS

Our primary interest is to develop and introduce new varieties with resistance to FHB, especially for SWS, a very important market class in PNW. Although the prediction accuracies were reasonably high under homogenous scenario, the scenario is far from practice. The future testing lines are more likely to be resistant lines from SWS market class. This study suggested that the current training population has reduced prediction accuracies when the testing population is restricted to either resistant lines, or the market class (**Figures 8**, **9**). Introducing lines in these categories and phenotyping them on multiple locations across years are critical to ensure the success of GS in breeding to improve FHB resistance.

The actual prediction accuracy should be better than reported in this study for scenarios where the testing populations contained less than 15 lines, such as the testing cohort that was restricted to SWS. Pearson correlation coefficient is downward biased in such case (Zhou et al., 2017). It is expected that larger training population leads to higher prediction accuracy. This was true for all cases in this study except the scenarios with testing population restricted to SWS for SEV and DON (**Figure 9**). As there were only 24 SWS lines, 20–50% of them as inference corresponded to that there were only 5–12 lines in the testing population. The smaller the proportion, the more downward biased on the Pearson correlation coefficient. After applying the unbiased correction with the Olkin and Pratt’s method (Olkin and Pratt, 1958), the reversed trend disappeared for SEV. For DON, all the averages of accuracies were below zero. The measurements of DON on the 24 SWS lines were not able to be predicted among themselves, or from the lines of the other two market classes.

## Conclusion

Correlation across environments were low for the three measurements of FHB: INC, SEV, and DON. Heritabilities of the means across the environments were 0.87, 0.68, and 0.44 for INC, SEV, and DON, respectively. Among the 170 lines, randomly selecting 90% as training population had accuracy of 0.63, 0.43, and 0.42 to predict the rest of 10% as testing population for INC, SEV, and DON, respectively. When testing population were restricted to resistant lines, or the wheat market class, substantial reductions of prediction accuracy were observed. Introducing new resistant soft white lines and phenotyping them in multiple environments would benefit GS for developing new wheat varieties with resistance to FHB.

## Ethics Statement

Any opinions, findings, conclusions, or recommendations expressed in this publication are those of the authors and do not necessarily reflect the views of the funding agencies. All datasets analyzed herein were published previously. This study did not involve samples from humans or animals.

## Author Contributions

ZZ and JC conceptualized the study with assistance from MP. HD performed the data analyses with assistance from RW and advice from YY and MP. RW, JA, and JC conducted phenotypic assessment of FHB resistance. RW and JC generated genotypic data through service from two USDA-ARS genotyping centers. ZZ and JC wrote the manuscript. All the authors revised the manuscript.

## Conflict of Interest Statement

The authors declare that the research was conducted in the absence of any commercial or financial relationships that could be construed as a potential conflict of interest.

## Footnotes

1 https://triticeaetoolbox.org/wheat/
