# Supplementary material for: Evaluation of the Potential for Genomic Selection to Improve Spring Wheat Resistance to Fusarium Head Blight in the Pacific Northwest
Source: Front Plant Sci. 2018 Jul 3;9:911. doi: 10.3389/fpls.2018.00911 (PMC6037981; doi:10.3389/fpls.2018.00911)
Supplement: Supplementary file 2 [file Table_2.PDF]

Table S2. Comparison of heritability estimations from raw and scaled kinship*

Trait by environment	Raw kinship	Scaled kinship	Trait by environment	Raw kinship	Scaled kinship
StP15_INC	0.195	0.277	GH16_SEV	0.259	0.356
StP16_INC	0.188	0.268	BLUP_SEV	0.569	0.677
AB15_INC	0.706	0.792	MEAN_SEV	0.569	0.677
AB16_INC	0.978	0.986	StP15_DON	0.329	0.438
BLUP_INC	0.803	0.866	CrK15_DON	0.258	0.356
MEAN_INC	0.803	0.866	AB15_DON	0.174	0.250
StP15_SEV	0.375	0.488	StP16_DON	0.485	0.599
StP16_SEV	0.666	0.760	BLUP_DON	0.333	0.442
AB16_SEV	0.252	0.348	MEAN_DON	0.331	0.439
GH15_SEV	0.283	0.385			

*The raw kinship was calculated from 10,101 SNPs for 170 spring wheat lines by using VanRaden algorithm implemented in GAPIT. The scaled kinship was transformed from the raw kinship by using the transformation in GAPIT to form pedigree-like kinship. The diagonals were 2 and the minimum elements equaled 0 for the least unrelated two inbred lines. The three FHB-related traits studied were incidence (INC), severity (SEV), and deoxynivalenol concentration (DON). Heritability was estimated for these traits under each environment, and with the mean and Best Linear Unbiased Prediction (BLUP) across the environments. Environment was defined as the combination of location and year. The four locations were Saint Paul, MN (StP), Crookston, MN (CrK), Aberdeen, ID (AB), and one greenhouse (GH), studied over two years (2015 and 2016).
