# Supplementary material for: Evaluation of the Potential for Genomic Selection to Improve Spring Wheat Resistance to Fusarium Head Blight in the Pacific Northwest
Source: Front Plant Sci. 2018 Jul 3;9:911. doi: 10.3389/fpls.2018.00911 (PMC6037981; doi:10.3389/fpls.2018.00911)
Supplement: Supplementary file 3 [file Table_3.PDF]

Table S3. Prediction accuracies for three FHB traits under different proportions of cross-validations*

Trait	10%	20%	30%	40%	50%	60%	70%	80%	90%
INC	0.630 (0.005)	0.626 (0.003)	0.621 (0.002)	0.607 (0.002)	0.594 (0.002)	0.568 (0.002)	0.534 (0.002)	0.488 (0.002)	0.382 (0.004)
SEV	0.434 (0.006)	0.409 (0.004)	0.391 (0.003)	0.363 (0.003)	0.335 (0.003)	0.286 (0.003)	0.231 (0.003)	0.161 (0.004)	0.058 (0.004)
DON	0.419 (0.006)	0.411 (0.004)	0.378 (0.003)	0.359 (0.003)	0.311 (0.003)	0.258 (0.003)	0.189 (0.004)	0.132 (0.004)	0.077 (0.003)

*The prediction accuracy is illustrated as the mean value, with the standard error in brackets. The three FHB traits studied were incidence (INC), severity (SEV), and deoxynivalenol concentration (DON). The cross-validation was performed by randomly selecting 10% to 90% from the 170 lines as the testing population. The rest of the lines were used as the training population. The prediction accuracy was calculated as the Pearson correlation between the observed and the predicted phenotypes.
