# Supplementary material for: Evaluation of the Potential for Genomic Selection to Improve Spring Wheat Resistance to Fusarium Head Blight in the Pacific Northwest
Source: Front Plant Sci. 2018 Jul 3;9:911. doi: 10.3389/fpls.2018.00911 (PMC6037981; doi:10.3389/fpls.2018.00911)
Supplement: Supplementary file 4 [file Table_4.PDF]

Table S4. Prediction accuracies of FHB traits when different proportions of FHB-resistant or FHB-susceptible lines were used as the testing population*

Type	Trait	10%	20%	30%	40%	50%	60%	70%	80%	90%	100%
Resistant	INC	0.528 (0.009)	0.539 (0.005)	0.543 (0.004)	0.532 (0.004)	0.522 (0.003)	0.512 (0.003)	0.497 (0.003)	0.49 (0.002)	0.478 (0.002)	0.492 (0)
	SEV	0.403 (0.011)	0.414 (0.006)	0.42 (0.005)	0.411 (0.004)	0.398 (0.003)	0.386 (0.003)	0.368 (0.002)	0.346 (0.002)	0.312 (0.002)	0.293 (0)
	DON	0.249 (0.013)	0.266 (0.008)	0.266 (0.006)	0.258 (0.004)	0.251 (0.004)	0.232 (0.004)	0.214 (0.003)	0.201 (0.003)	0.187 (0.002)	0.172 (0)
Susceptible	INC	0.673 (0.007)	0.678 (0.004)	0.681 (0.003)	0.667 (0.003)	0.66 (0.002)	0.638 (0.002)	0.616 (0.002)	0.585 (0.002)	0.548 (0.002)	0.52 (0)
	SEV	0.373 (0.009)	0.358 (0.006)	0.339 (0.004)	0.317 (0.004)	0.296 (0.003)	0.265 (0.003)	0.23 (0.003)	0.179 (0.003)	0.104 (0.003)	0.023 (0)
	DON	0.522 (0.007)	0.509 (0.005)	0.493 (0.004)	0.472 (0.003)	0.441 (0.003)	0.394 (0.004)	0.331 (0.004)	0.267 (0.004)	0.143 (0.005)	-0.082 (0)

*The prediction accuracy is presented as the mean value, with the standard error in brackets. The 170 wheat lines were classified into FHB-resistant (69) and FHB-susceptible (101) subpopulations. The subpopulation was randomly sampled as the testing population with proportions of 10% to 100%. The three FHB traits studied were incidence (INC), severity (SEV), and deoxynivalenol concentration (DON). The prediction accuracy was calculated as the Pearson correlation between the observed and the predicted phenotypes.
