# Supplementary material for: Evaluation of the Potential for Genomic Selection to Improve Spring Wheat Resistance to Fusarium Head Blight in the Pacific Northwest
Source: Front Plant Sci. 2018 Jul 3;9:911. doi: 10.3389/fpls.2018.00911 (PMC6037981; doi:10.3389/fpls.2018.00911)
Supplement: Supplementary file 5 [file Table_5.PDF]

Table S5. Prediction accuracies of FHB traits when different proportions of market class lines were used as the testing population*

Market	Trait	10%	20%	30%	40%	50%	60%	70%	80%	90%	100%
HRS	INC	0.657 (0.009)	0.689 (0.004)	0.681 (0.003)	0.696 (0.003)	0.694 (0.002)	0.697 (0.002)	0.695 (0.001)	0.694 (0.001)	0.682 (0.001)	0.672 (0)
	SEV	0.425 (0.012)	0.425 (0.007)	0.395 (0.005)	0.37 (0.004)	0.331 (0.004)	0.284 (0.004)	0.231 (0.004)	0.175 (0.004)	0.087 (0.004)	-0.027 (0)
	DON	0.489 (0.01)	0.464 (0.006)	0.447 (0.005)	0.428 (0.004)	0.392 (0.004)	0.355 (0.004)	0.323 (0.004)	0.278 (0.003)	0.245 (0.003)	0.226 (0)
HWS	INC	0.583 (0.01)	0.631 (0.005)	0.611 (0.004)	0.613 (0.003)	0.594 (0.003)	0.587 (0.003)	0.561 (0.002)	0.531 (0.002)	0.489 (0.002)	0.419 (0)
	SEV	0.35 (0.01)	0.335 (0.007)	0.298 (0.005)	0.282 (0.004)	0.248 (0.004)	0.214 (0.004)	0.17 (0.003)	0.121 (0.003)	0.06 (0.002)	-0.011 (0)
	DON	0.493 (0.01)	0.513 (0.006)	0.511 (0.004)	0.499 (0.003)	0.468 (0.003)	0.433 (0.003)	0.375 (0.003)	0.301 (0.004)	0.187 (0.004)	0.053 (0)
SWS	INC		0.349 (0.012)	0.343 (0.01)	0.328 (0.007)	0.283 (0.007)	0.273 (0.006)	0.219 (0.005)	0.193 (0.005)	0.169 (0.003)	0.167 (0)
	SEV		0.335 (0.014)	0.396 (0.011)	0.447 (0.008)	0.458 (0.007)	0.472 (0.006)	0.484 (0.005)	0.463 (0.006)	0.408 (0.006)	0.262 (0)
	DON		-0.22 (0.015)	-0.227 (0.012)	-0.205 (0.009)	-0.19 (0.007)	-0.184 (0.006)	-0.144 (0.005)	-0.128 (0.004)	-0.09 (0.002)	-0.079 (0)

*The prediction accuracy is illustrated as the mean value, with the standard error in brackets. The market classes studied were Hard Red Spring (HRS), Hard White Spring (HWS), and Soft White Spring (SWS). For each market class, 10% to 100% were randomly removed and used as the testing population; the rest of the lines, including the lines of the other market classes, were used as the training population. The three FHB traits studied were incidence (INC), severity (SEV), and deoxynivalenol concentration (DON). The prediction accuracy was calculated as the Pearson correlation between the observed and the predicted phenotypes. The combination of 10% and SWS was not available because, in this scenario, only two lines were in the testing population. The Pearson correlation would be either -1 or 1.
