# Supplementary material for: Evaluation of the Potential for Genomic Selection to Improve Spring Wheat Resistance to Fusarium Head Blight in the Pacific Northwest
Source: Front Plant Sci. 2018 Jul 3;9:911. doi: 10.3389/fpls.2018.00911 (PMC6037981; doi:10.3389/fpls.2018.00911)
Supplement: Supplementary file 6 [file Table_6.PDF]

Table S6. Prediction accuracies of FHB traits when different proportions of lines within the same market class were used as the testing population*

Market	Trait	10%	20%	30%	40%	50%	60%	70%	80%	90%
HRS	INC	0.633 (0.009)	0.645 (0.005)	0.636 (0.004)	0.624 (0.003)	0.611 (0.003)	0.589 (0.003)	0.534 (0.004)	0.468 (0.005)	0.331 (0.007)
	SEV	0.364 (0.012)	0.365 (0.007)	0.336 (0.006)	0.293 (0.005)	0.274 (0.005)	0.229 (0.005)	0.193 (0.005)	0.142 (0.005)	0.078 (0.006)
	DON	0.367 (0.012)	0.347 (0.007)	0.328 (0.005)	0.308 (0.005)	0.294 (0.004)	0.256 (0.004)	0.215 (0.004)	0.187 (0.005)	0.12 (0.006)
HWS	INC	0.6 (0.009)	0.597 (0.005)	0.564 (0.004)	0.531 (0.004)	0.493 (0.004)	0.453 (0.004)	0.37 (0.004)	0.269 (0.005)	0.103 (0.007)
	SEV	0.358 (0.009)	0.332 (0.006)	0.316 (0.004)	0.281 (0.004)	0.238 (0.004)	0.195 (0.004)	0.14 (0.004)	0.085 (0.004)	0.034 (0.004)
	DON	0.448 (0.01)	0.464 (0.006)	0.449 (0.005)	0.425 (0.004)	0.386 (0.004)	0.35 (0.004)	0.302 (0.004)	0.228 (0.004)	0.145 (0.005)
SWS	INC		0.578 (0.011)	0.513 (0.011)	0.367 (0.01)	0.268 (0.01)	0.185 (0.009)	0.049 (0.008)		
	SEV		0.491 (0.011)	0.492 (0.008)	0.457 (0.007)	0.432 (0.008)	0.402 (0.008)	0.317 (0.011)		
	DON		-0.255 (0.014)	-0.274 (0.01)	-0.252 (0.008)	-0.219 (0.007)	-0.188 (0.006)	-0.128 (0.005)		

*The prediction accuracy is illustrated as the mean value, with the standard error in brackets. The market classes studied were Hard Red Spring (HRS), Hard White Spring (HWS), and Soft White Spring (SWS). For each market class, 10% to 100% were randomly removed as the testing population; the rest of the lines within the same class were used as the training population. The three traits studied were incidence (INC), severity (SEV), and deoxynivalenol concentration (DON). The prediction accuracy was calculated as the Pearson correlation between the observed and the predicted phenotypes. The combinations of 10%, 80%, and 90% in SWS were not available because, in these scenarios, only a few lines were in the testing or training populations.
