# Supplementary material for: Evaluation of the Potential for Genomic Selection to Improve Spring Wheat Resistance to Fusarium Head Blight in the Pacific Northwest
Source: Front Plant Sci. 2018 Jul 3;9:911. doi: 10.3389/fpls.2018.00911 (PMC6037981; doi:10.3389/fpls.2018.00911)
Supplement: Supplementary file 7 [file Image_1.PDF]

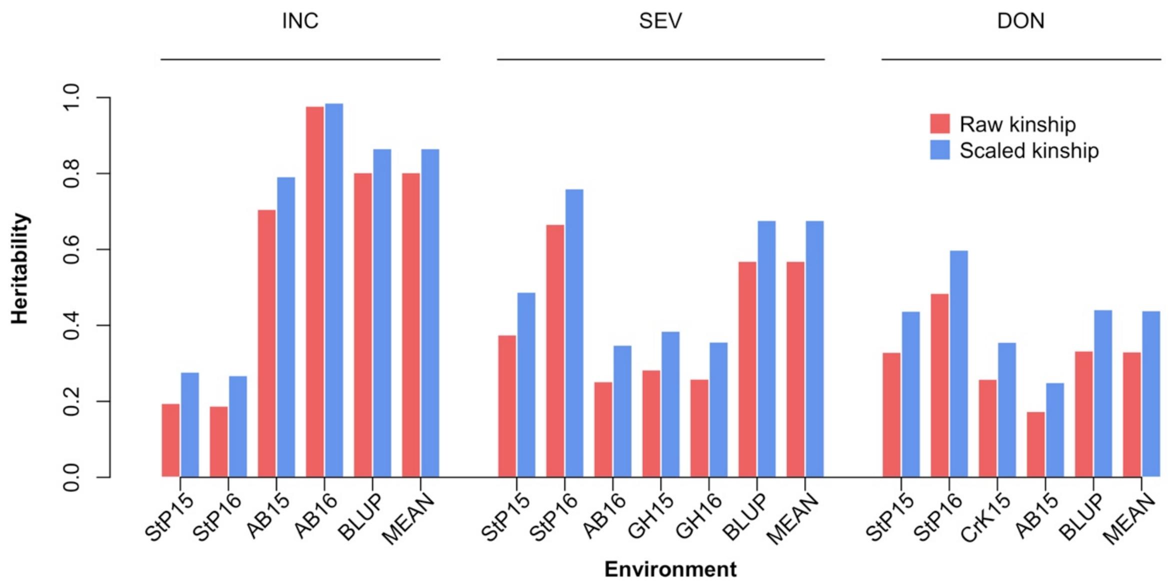

Figure S1. Comparison of heritability estimation from raw and scaled kinship. The raw kinship was calculated from 10,101 SNPs for 170 spring wheat lines by using VanRaden algorithm, implemented in GAPIT. The scaled kinship was transformed from the raw kinship by using the transformation in GAPIT to form pedigree-like kinship. The diagonals were 2 and the minimum elements equaled 0 for the least unrelated two inbred lines. The three FHB traits studied were incidence (INC), severity (SEV), and deoxynivalenol concentration (DON). Heritability was estimated for these traits under each environment and with the mean and Best Linear Unbiased Prediction (BLUP) across the environments. Environment was defined as the combination of location and year. The four locations were Saint Paul, MN (StP), Crookston, MN (CrK), Aberdeen, ID (AB), and one greenhouse (GH), studied over two years (2015 and 2016).
