# Supplementary material for: Evaluation of the Potential for Genomic Selection to Improve Spring Wheat Resistance to Fusarium Head Blight in the Pacific Northwest
Source: Front Plant Sci. 2018 Jul 3;9:911. doi: 10.3389/fpls.2018.00911 (PMC6037981; doi:10.3389/fpls.2018.00911)
Supplement: Supplementary file 8 [file Image_2.PDF]

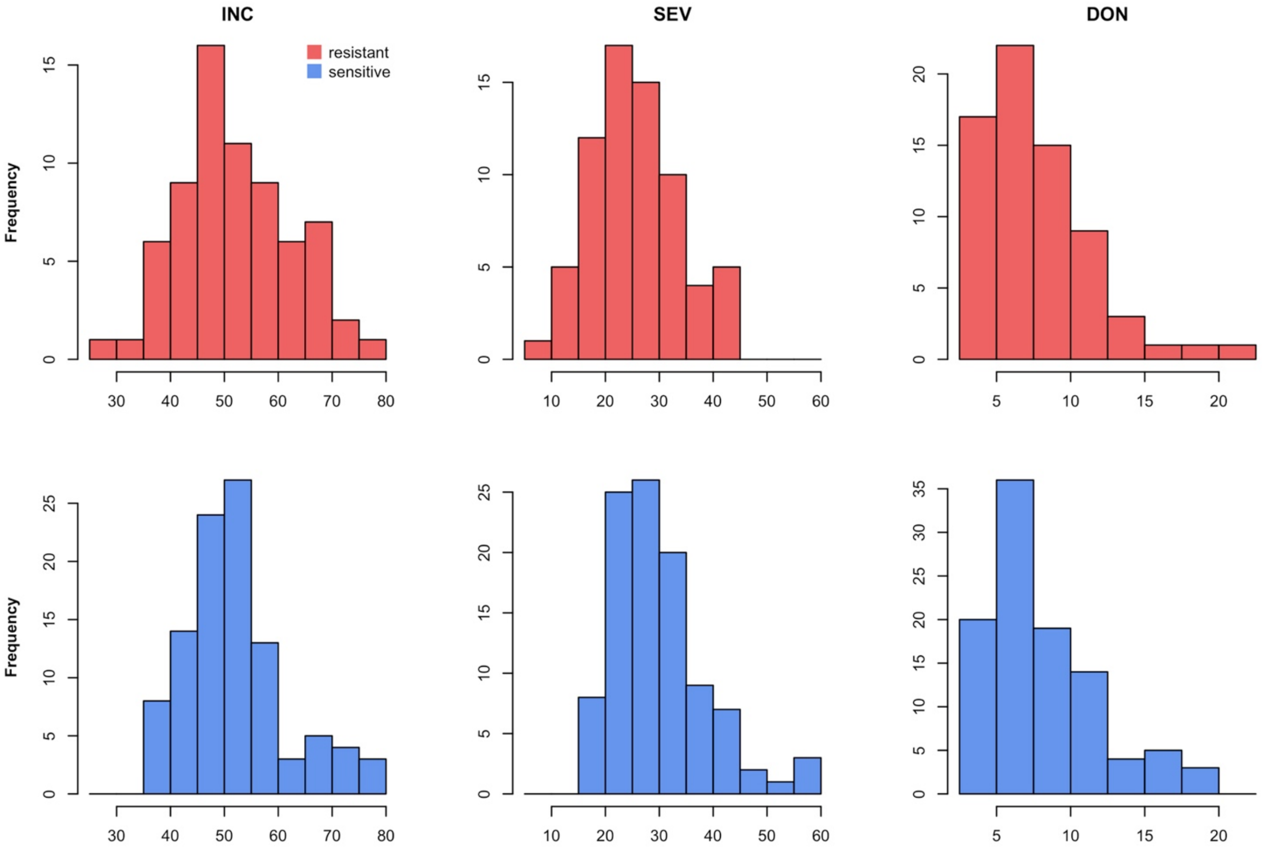

Figure S2. Distributions of FHB-related traits categorized by resistant and susceptible groups. The 170 spring wheat lines were classified into resistant (69 lines) and susceptible (101 lines) subgroups according to the preference of the breeding programs in Washington and Idaho. The three FHB traits studied were incidence (INC), severity (SEV), and deoxynivalenol concentration (DON). Mean values are presented.
