# Supplementary material for: Evaluation of the Potential for Genomic Selection to Improve Spring Wheat Resistance to Fusarium Head Blight in the Pacific Northwest
Source: Front Plant Sci. 2018 Jul 3;9:911. doi: 10.3389/fpls.2018.00911 (PMC6037981; doi:10.3389/fpls.2018.00911)
Supplement: Supplementary file 9 [file Image_3.PDF]

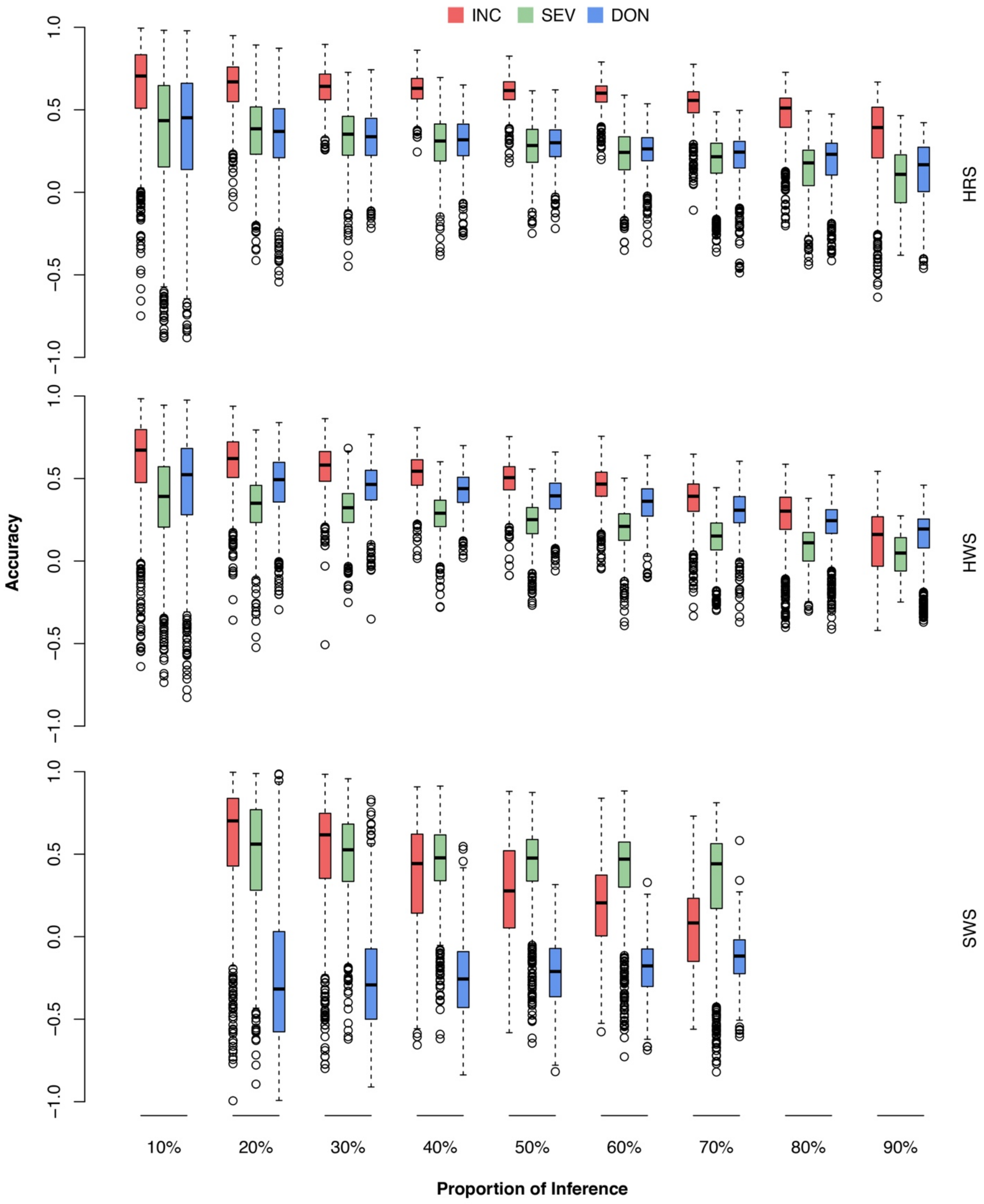

Figure S3. Prediction accuracies of FHB traits when different proportions of lines within the same wheat market class were used as the testing population. The wheat market classes studied were Hard Red Spring (HRS), Hard White Spring (HWS), and Soft White Spring (SWS). For each market class, 10% to 100% were randomly removed as the testing population, the rest of the lines within the same class were used as training population. The three traits studied were incidence (INC), severity (SEV), and deoxynivalenol concentration (DON). The prediction accuracy was calculated as

the Pearson correlation between the observed and the predicted phenotypes. The combinations of 10%, 80%, and 90% in SWS were not available because, in these scenarios, only a few lines were in the testing or training populations.
